# Supplementary material for: Saccharomyces boulardii (CNCM I-745) improves intestinal damage in sepsis by remodeling intestinal flora structure
Source: Front Cell Infect Microbiol. 2025 Jul 31;15:1602792. doi: 10.3389/fcimb.2025.1602792 (PMC12350329; doi:10.3389/fcimb.2025.1602792)
Supplement: Supplementary file 1 [file DataSheet1.zip › summary/summary/6_Taxonomy_community/1_Taxonomy_profiling/1_Domain/Group/Domain_abund_top30.Group.stacked_bar.pdf]

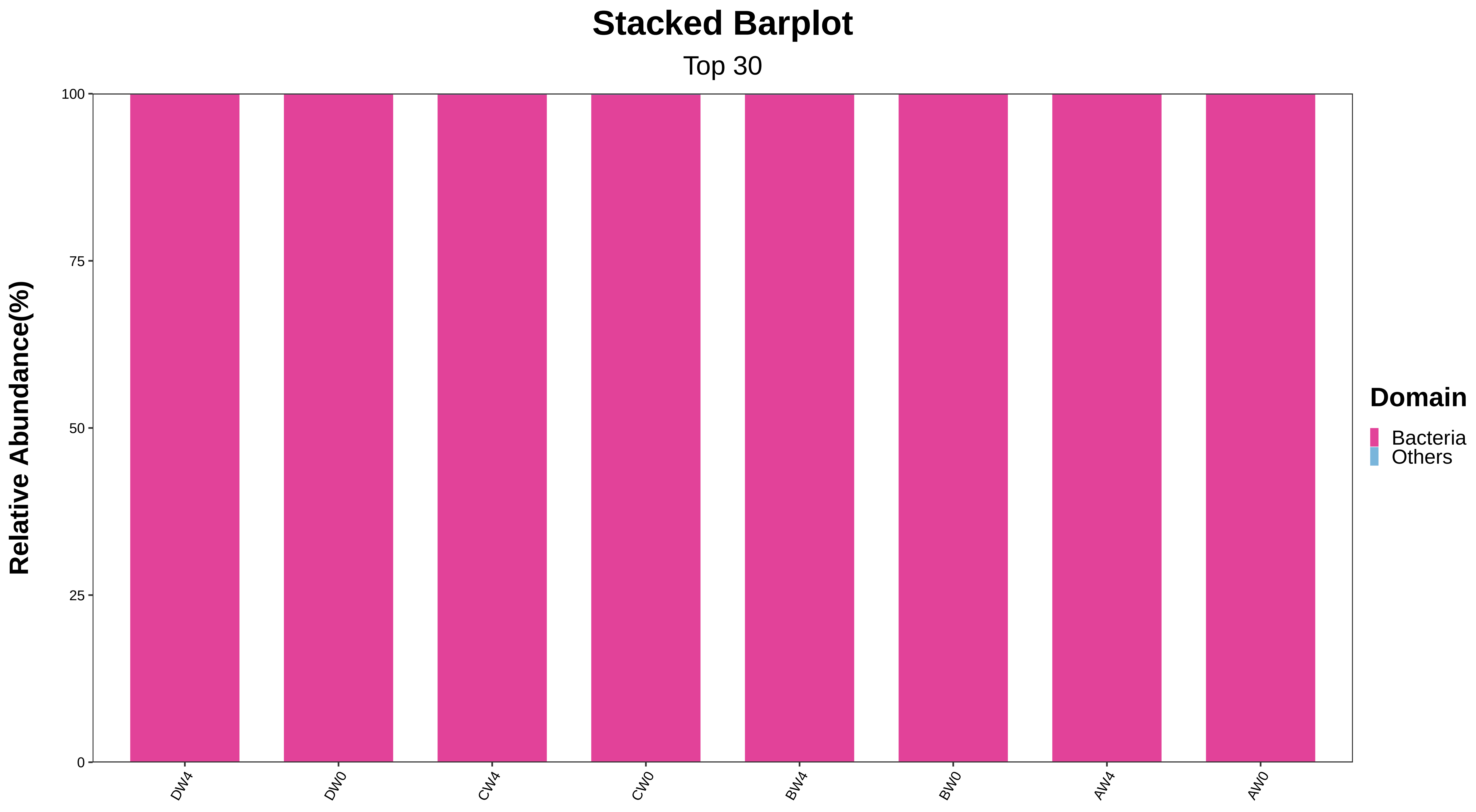

Stacked Barplot
Top 30
Relative Abundance(%)
100
75
50
25
0
DW4
DW0
CW4
CW0
BW4
BW0
AW4
AW0
Domain
Bacteria
Others
